# Arterial stiffness and incident chronic kidney disease: a large population-based cohort study

**DOI:** 10.1007/s40620-024-01968-x

**Published:** 2024-05-29

**Authors:** Angela Beros, John Sluyter, Alun Hughes, Bernhard Hametner, Siegfried Wassertheurer, Robert Scragg

**Affiliations:** 1 School of Population Health, University of Auckland, Auckland, New Zealand; 2 Institute of Cardiovascular Sciences, University College London, London, UK; 3 Center for Health and Bioresources, AIT Austrian Institute of Technology, Vienna, Austria

**Keywords:** Arterial stiffness, Cohort study, Chronic kidney disease, Pulse wave velocity, Pulse pressure

## Abstract

**Background/aims:**

Evidence from large population-based cohorts as to the association of arterial stiffness and incident chronic kidney disease (CKD) is mixed. This large population-based study aimed to investigate whether arterial stiffness, assessed oscillometrically, was associated with incident CKD.

**Methods:**

The study population comprised 4838 participants from the Vitamin D Assessment (ViDA) Study without known CKD (mean ± SD age = 66 ± 8). Arterial stiffness was assessed from 5 April, 2011 to 6 November, 2012 by way of aortic pulse wave velocity, estimated carotid-femoral pulse wave velocity, and aortic pulse pressure. Incident CKD was determined by linkage to national hospital discharge registers. Cox proportional hazards regression was used to assess the risk of CKD in relation to chosen arterial stiffness measures over the continuum and quartiles of values.

**Results:**

During a mean ± SD follow-up of 10.5 ± 0.4 years, 376 participants developed incident CKD. Following adjustment for potential confounders, aortic pulse wave velocity (hazard ratio (HR) per SD increase 1.69, 95% CI 1.45–1.97), estimated carotid-femoral pulse wave velocity (HR per SD increase 1.84, 95% CI 1.54–2.19), and aortic pulse pressure (HR per SD increase 1.37, 95% CI 1.22–1.53) were associated with the incidence of CKD. The risk of incident CKD was, compared to the first quartile, higher in the fourth quartile of aortic pulse wave velocity (HR 4.72, 95% CI 2.69–8.27; *P*_trend_ < 0.001), estimated carotid-femoral pulse wave velocity (HR 4.28, 95% CI 2.45–7.50; *P*_trend_  < 0.001) and aortic pulse pressure (HR 2.71, 95% CI 1.88–3.91; *P*_trend_  < 0.001).

**Conclusions:**

Arterial stiffness, as measured by aortic pulse wave velocity, estimated carotid-femoral pulse wave velocity, and aortic pulse pressure may be utilised in clinical practice to help identify people at risk of future CKD.

**Trial registration:**

www.anzctr.org.au identifier:ACTRN12611000402943.

**Graphical abstract:**

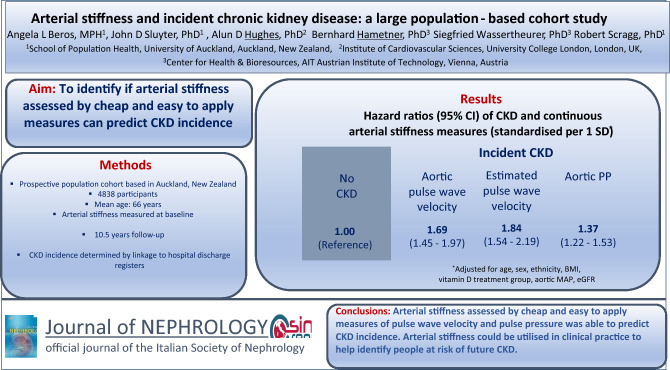

**Supplementary Information:**

The online version contains supplementary material available at 10.1007/s40620-024-01968-x.

## Introduction

Chronic kidney disease (CKD) can lead to disability and is associated with premature death, particularly due to cardiovascular factors. A person is said to have CKD when their kidneys have had abnormal structure and function for > 3 months [1]. It was estimated that in 2017 11% of individuals worldwide (> 840 million people) were living with CKD [2]. Kidney function can decline by up to 90% before symptoms develop [3]. Chronic kidney disease is also largely irreversible, however, kidney function can often be preserved if the condition is properly managed. The prevalence of CKD increases with age and tends to be more common in ethnic minorities. In New Zealand, approximately 12% of the population may have CKD, with the highest prevalence estimated to be in indigenous Māori, Pacific (in New Zealand, predominantly people from the smaller Pacific nations of Samoa, Tonga and the Cook Islands) and people from South-Asia (the Indian Subcontinent)[4].

Arterial stiffness describes the rigidity of arterial walls and primarily refers to the stiffness of larger arteries and the aorta. Pulse wave velocity, a measure of the speed at which a forward pressure wave propagates in an artery is a measure of arterial stiffness, with higher measures indicating increased arterial rigidity [5]. The gold standard for evaluating aortic stiffness is the measurement of carotid-femoral pulse wave velocity [6]. Aortic pulse wave velocity can also be estimated from a blood pressure (BP) waveform obtained from a more peripheral artery, often the radial or brachial artery [6, 7]. Aortic pulse pressure is also recognised as a rough estimate of arterial stiffness [6].

Arterial stiffness may contribute to CKD incidence and progression due to increased pulsatile pressure on kidney microvasculature [5]. The evidence from large cohort studies assessing arterial stiffness and incident CKD, however, is inconclusive. Various studies, including the *Framingham Heart Study* [8] and the *Health ABC Study* [9] have found an association of arterial stiffness and incident CKD [10, 11]. Other such studies, however, have not observed a significant relationship [12, 13]. Four of these studies measured carotid-femoral pulse wave velocity by way of Doppler or tonometry [8, 9, 12, 13]. These techniques require specialized training, involve measurement from two places on the body whilst the participant lies supine, and may utilise bulky equipment. Two studies employed other measures of pulse wave velocity, namely brachial-ankle pulse wave velocity [10] and heart-ankle pulse wave velocity [11], which though easier to obtain than carotid-femoral pulse wave velocity still require the application of cuffs to all four limbs.

For arterial stiffness to be employed in clinical practice, it must be easy to measure, use a method that is acceptable to patients, and be cost-effective [14]. Technological advancements have led to the development of oscillometric devices which compute aortic pulse wave velocity using the measured brachial artery pressure waveform [6]; these devices are affordable, portable, and user-friendly as they mirror home BP devices and only require one cuff. Given that both age and mean arterial pressure influence pulse wave velocity, equations have also been formulated to estimate carotid-femoral pulse wave velocity [15].

We aimed to assess whether arterial stiffness, as assessed oscillometrically by aortic pulse wave velocity, estimated carotid-femoral pulse wave velocity, or aortic pulse pressure predicted incident CKD in a large population-based cohort, aged 50–84 years, in Auckland, New Zealand.

## Materials and methods

### Participants

We analysed data from the Vitamin D Assessment (ViDA) Study, a randomised controlled trial with the primary aim of evaluating the efficacy of vitamin D supplementation on reducing the incidence of cardiovascular disease (CVD)—no effect was observed [16]. Ethics approval was granted by the Ministry of Health (MoH) Multi-region Ethics committee (MEC/09/08/082). Participants provided written informed consent. Full study details have been published elsewhere [17].

Between 5 April, 2011, and 6 November, 2012, 5250 participants primarily enlisted from 55 family practices located in Auckland, New Zealand underwent a baseline assessment at the University of Auckland, with 5108 participants included in the final study.

We excluded from the analysis 203 participants for whom arterial stiffness measures were missing or excluded (due to poor quality waveforms with a signal-to-noise ratio of < 6 dB). We further excluded 67 participants identified as having CKD for the period between 1 April 2009 and each participant’s baseline interview, resulting in 4838 participants being included in the final analysis.

### CKD and follow-up

The MoH allocates New Zealand residents a National Health Index number. This number was used to track deaths and hospital discharges [utilising the International Statistical Classification of Diseases and Health Related Problems, Tenth Revision (ICD-10) coding system]. Incident CKD was identified from the first occurrence of any hospital discharge record with the relevant ICD-10 code for the period beginning with each participant’s baseline interview up to 31 August, 2022. Such data were also used to exclude participants with CKD at baseline as identified from records for the period between 1 April, 2009 and each participant’s baseline interview, together with self-reported diabetic kidney disease at the baseline interview. The relevant ICD-10 codes are reported in Supplementary Information, Table S1.

### Arterial stiffness

Central  BP measurements including heart rate, aortic pulse pressure and pulse waveform characteristics were obtained using a BP+ monitor (Uscom, Sydney, Australia), an oscillometric device with a correctly sized cuff placed on the upper left arm. The BP+ monitor has shown good intra-test and inter-test reliability when measuring central systolic BP [18]. The ARCSolver pulse wave algorithm (AIT Austrian Institute of Technology, GmbH, Vienna, Austria) was used to calculate aortic pulse wave velocity using the pressure waveform. This algorithm has been validated against aortic pulse wave velocity measured with an intra-aortic catheter [7]. Oscillometric devices can have inbuilt pulse wave algorithms, and hence are able to generate and save waveform parameters, including aortic pulse wave velocity, at the time of capture. Aortic mean arterial pressure was calculated as the area under the aortic pressure waveform (mmHg*seconds)/cardiac cycle duration (seconds) [19].

We implemented equations for estimated carotid-femoral pulse wave velocity based on those put forward by Greve et al. [20] and which are based on the Reference Values for Arterial Stiffness Collaboration equations [15]. These equations have predicted CVD events [20] and incident diabetes [21]. For participants with one or more CVD risk factors at baseline, estimated carotid-femoral pulse wave velocity was calculated as 4.62–0.13 × age + 0.0018 × age squared + 0.0006 × age × brachial mean arterial pressure (bMAP) + 0.0284 × bMAP. For participants with no CVD risk factors at baseline, estimated carotid-femoral pulse wave velocity was calculated as 9.587 − 0.402 × age + 0.004560 × age squared − 0.000026 × age squared × bMAP + 0.003176 × age × bMAP-0.018322 × bMAP. As defined in the development of these equations, bMAP was calculated as brachial diastolic BP + 0.4 × brachial pulse pressure. Cardiovascular disease risk factors, with criteria based on those used to develop the original equations were current tobacco smoking, hypertension (brachial systolic BP ≥ 140, or brachial diastolic BP ≥ 90, or as reported by a physician), dyslipidaemia (total cholesterol > 4.9 mmol/L, or HDL-C < 1 mmol/L, or total cholesterol/HDL-C ratio < 4, or as reported by a physician) and previous CVD events (heart attack, angina, heart failure, irregular heartbeat, transient ischaemic attack, stroke, carotid artery stenosis, or intermittent claudication as reported by a physician, or self-report of pacemaker).

### Covariates

The baseline assessment included a questionnaire administered by trained interviewers adhering to a standardised protocol. Information was collected on socio-demographic factors (age, gender, self-reported ethnicity), lifestyle factors (divided into two categories: current and never/former for tobacco smoking and alcohol consumption, and > 2 h per week and ≤ 2 h per week for vigorous physical activity), medical history as reported by a physician (including diabetes, high cholesterol, hypertension and cardiovascular disease), current prescribed medications (diabetic, antihypertensives and lipid-lowering) and self-administered supplements, including vitamin D.

Participants were further identified as having diabetes or prediabetes if, between 1 April, 2009 and their baseline interview, they were identified through their National Health Index number as having an ICD-10 Code relating to diabetes or having received a prescription for diabetes medication. The relevant ICD-10 codes are reported in Supplementary Information, Table S1.

Socioeconomic status was estimated using a participant’s residential neighbourhood decile, as classified by the New Zealand Index of Deprivation 2013 (NZDep2013) (ranging from 1 to 10, with 1 representing the least deprived areas). Height was measured to the nearest 0.1 cm and weight to the nearest 0.1 kg. Body mass index (BMI) was calculated as weight (kg)/height (m^2^).

A non-fasting blood sample was collected and stored at a temperature of − 80 °C with samples subsequently analysed for total cholesterol, high-density lipoprotein cholesterol (HDL-C) and serum creatinine. Estimated glomerular filtration rate (eGFR) was calculated according to the Chronic Kidney Disease Epidemiology Collaboration Equation [22].

### Statistical analysis

Data were analysed collectively and were also divided into aortic pulse wave velocity quartiles. The characteristics of the study population, by incident CKD status and by quartile of aortic pulse wave velocity were shown as mean ± SD or median (IQR) for continuous measures and frequency (%) for categorical measures.

The incidence rate of CKD for person-years was calculated as the number of incident cases/the total duration of follow-up (for each participant being the earlier of death date or 31 August, 2022). Comparisons of baseline characteristics, between participants who developed CKD and those who did not, were performed using *t* tests for continuous variables and Chi-square tests for categorical variables. We censored participants who died during the follow-up period.

Multivariable Cox regression models were used to evaluate the association between arterial stiffness as a continuous variable (measured by aortic pulse wave velocity, estimated carotid-femoral pulse wave velocity and aortic pulse pressure) and incident CKD. The analysis employed hazard ratios (HRs) with 95% CIs. We also computed HRs based on a SD increase of each arterial stiffness measure (sHR) and for quartiles of each arterial stiffness measure using the lowest level of each arterial stiffness measure as the reference group. Covariates were selected for inclusion in the models based on similar studies [9, 10, 13, 23–26].

Model 1 was adjusted for age, sex, ethnicity, BMI, eGFR, vitamin D treatment group and aortic mean arterial pressure. Model 2 comprised Model 1 plus lifestyle factors (tobacco smoking, alcohol drinking and vigorous physical activity), NZDep2013, history of CVD, diabetes/prediabetes, medications (diabetes, antihypertensives, lipid-lowering), total cholesterol, HDL-C and heart rate. Kaplan–Meier curves, stratified by quartiles of each arterial stiffness measure, were used to show the relationship between arterial stiffness and incident CKD.

SAS V9.4 (Cary, North Carolina, USA) was used for statistical analysis and all statistical tests were 2-sided.

### Sensitivity analysis

To assess the influence of participants with undiagnosed CKD at baseline, we performed a sensitivity analysis where, in addition to any participants with identified CKD at baseline, we also excluded any other participants who had an eGFR < 60 ml/min per 1.73 m^2^, as one of the criteria for diagnosing CKD is having this reading for > 3 months [1].

## Results

### Baseline characteristics

Baseline characteristics of the participants (*n* = 4838) are presented in Table 1, overall, by incident CKD status and by quartile of baseline aortic pulse wave velocity. Mean age was 66 ± 8 years and 58% were men. The majority (84%) were European/other ethnicity, 5% identified as Māori, 6% as Pacific and 5% as South Asian. Mean ± SD arterial stiffness at baseline, as measured by aortic pulse wave velocity was 9.50 ± 1.75 m/s [median (IQR): 9.38 (2.47)], estimated carotid-femoral pulse wave velocity, 10.97 ± 1.72 m/s and aortic pulse pressure, 68 ± 16 mmHg. Of those participants, 4802 (99%) had ≥ 1 CVD risk factor.

**Table 1:** Characteristics of participants without chronic kidney disease (CKD) at baseline in relation to the total sample, incident CKD status and quartile of baseline aortic pulse wave velocity (aPWV)

	Total sample	Incident CKD status			Quartile of aPWV, m/s			
		No CKD	CKD	P-Value	PWV < 8.20	8.20 ≤ PWV < 9.38	9.38 ≤ PWV < 10.67	PWV ≥ 10.67
Number, *n*	4838	4462	376		1209	1210	1209	1210
Age, y	66 ± 8	66 ± 8	71 ± 8	< 0.001	57 ± 4	64 ± 4	69 ± 4	76 ± 5
Men, %	2811 (58)	2545 (91)	266 (9)	< 0.001	591 (21)	682 (24)	742 (27)	796 (28)
Ethnicity, %				< 0.001				
European/other	4051 (84)	3776 (93)	275 (7)		808 (20)	1021 (25)	1092 (27)	1130 (28)
Māori	249 (5)	221 (89)	28 (11)		125 (50)	60 (24)	35 (14)	29 (12)
Pacific	301 (6)	251 (83)	50 (17)		147 (49)	74 (24)	54 (18)	26 (9)
South Asian	237 (5)	214 (90)	23 (10)		129 (54)	55 (23)	28 (12)	25 (11)
Tobacco smoker, current, %	303 (6)	280 (92)	23 (8)	< 0.001	132 (44)	77 (25)	58 (19)	36 (12)
Alcohol drinker, current, %	4186 (87)	3884 (93)	302 (7)	< 0.001	990 (24)	1051 (25)	1082 (26)	1063 (25)
Vigorous physical activity, > 2 h per week, %	2190 (45)	2075 (95)	115 (5)	< 0.001	602 (27)	620 (28)	515 (24)	453 (21)
Body mass index, kg/m^2 a^	28 ± 5	28 ± 5	31 ± 6	< 0.001	28 ± 6	29 ± 5	28 ± 5	28 ± 4
Diabetes/prediabetes, current, %^a^	552 (11)	381 (69)	171 (31)	< 0.001	123 (22)	133 (24)	150 (27)	146 (27)
Cardiovascular disease, current/past, %	1124 (23)	957 (85)	167 (15)	< 0.001	140 (13)	234 (21)	307 (27)	443 (39)
NZDep2013, deciles 6-10^a^	1640 (34)	1473 (90)	167 (10)	< 0.001	476 (29)	418 (25)	374 (23)	372 (23)
Medications								
Antihypertensives, %	1748 (36)	1498 (86)	250 (14)	< 0.001	240 (14)	394 (22)	500 (29)	614 (35)
Diabetic medications, %	512 (11)	356 (70)	156 (30)	< 0.001	126 (25)	119 (24)	141 (27)	126 (24)
Lipid lowering medications, %	1684 (35)	1473 (87)	211 (13)	< 0.001	277 (16)	420 (25)	480 (29)	507 (30)
Vitamin D supplementation, %	2422 (50)	2242 (93)	180 (7)	0.39	608 (25)	609 (25)	599 (25)	606 (25)
eGFR, ml/min per 1.73 m^2^	72 ± 12	73 ± 11	61 ± 14	< 0.001	77 ± 11	73 ± 11	71 ± 11	66 ± 11
Total cholesterol, mmol/L	4.87 ± 1.09	4.92 ± 1.08	4.21 ± 1.06	< 0.001	5.03 ± 1.09	4.93 ± 1.09	4.84 ± 1.07	4.66 ± 1.09
HDL-C, mmol/L	1.42 ± 0.43	1.43 ± 0.42	1.24 ± 0.41	< 0.001	1.42 ± 0.42	1.42 ± 0.43	1.42 ± 0.42	1.41 ± 0.43
aPWV, m/s, median (IQR)	9.38 (2.47)	9.30 (2.41)	10.51 (2.63)	< 0.001	7.46 (0.89)	8.80 (0.61)	9.95 (0.65)	11.70 (1.34)
ecfPWV, m/s	10.97 ± 1.72	10.89 ± 1.71	11.91 ± 1.67	< 0.001	8.96 ± 0.78	10.32 ± 0.59	11.43 ± 0.61	13.18 ± 0.98
Systolic BP, aortic, mmHg	140 ± 19	140 ± 19	145 ± 19	< 0.001	127 ± 14	136 ± 15	144 ± 16	155 ± 20
Diastolic BP, aortic, mmHg	72 ± 6	72 ± 6	72 ± 6	0.91	71 ± 6	72 ± 6	73 ± 6	74 ± 7
Pulse pressure, aortic, mmHg	68 ± 16	68 ± 15	73 ± 17	< 0.001	56 ± 11	64 ± 11	71 ± 12	81 ± 16
Mean arterial pressure, aortic, mmHg	100 ± 12	100 ± 12	99 ± 12	0.70	95 ± 10	98 ± 11	101 ± 12	105 ± 12
Heart rate, bpm	63 ± 10	63 ± 10	65 ± 11	0.002	65 ± 10	63 ± 10	63 ± 10	62 ± 10

Data shown as mean ± SD for continuous measures and frequency (column % for total sample; row % for incident CKD status and quartile of aPWV) for categorical measures. BP, blood pressure; ecfPWV, estimated carotid-femoral PWV; PWV, Pulse wave velocity; HDL-C high-density lipoprotein cholesterol; NZDep2013, New Zealand Index of Deprivation 2013

### Incident CKD

The mean ± SD length of follow up was 10.5 ± 0.4 years, during which 376 participants developed CKD, with a higher cumulative incidence for Māori (11%), Pacific (17%) and South Asian (10%), compared to European/other (7%) (Table 1).

Table 2 shows associations between measures of arterial stiffness and risk of incident CKD both overall, and by quartile of each arterial stiffness measure. The incidence of CKD overall was 7.38 per 1000 person years.

**Table 2:** Incidence and hazard ratios of chronic kidney disease (CKD) in relation to the total sample and quartiles of baseline arterial stiffness measures

Participants, *n*^c^	Total sample^a^	Total sample^b^	Quartile of arterial stiffness—hazard ratios (95% CI)				*P* for trend
	4838		1209	1210	1209	1210	
aPWV, m/s			PWV < 8.20	8.20 ≤ PWV < 9.38	9.38 ≤ PWV < 10.67	PWV ≥ 10.67	
CKD incidence, *n* (*n* per 1000 person years)	376 (7.38)		37 (2.85)	63 (4.95)	102 (8.09)	174 (13.78)	
Model 1	1.35 (1.24–1.48)	1.69 (1.45–1.97)	Reference	1.79 (1.15–2.79)	3.07 (1.83–5.15)	4.72 (2.69–8.27)	< 0.001
Model 2	1.31 (1.19–1.44)	1.60 (1.36–1.90)	Reference	1.71 (1.08–2.72)	2.68 (1.56–4.61)	4.19 (2.31–7.59)	< 0.001
ecfPWV, m/s			PWV < 9.71	9.71 ≤ PWV < 10.87	10.87 ≤ PWV < 12.11	PWV ≥ 12.11	
CKD incidence, *n* (*n* per 1000 person years)	376 (7.38)		38 (2.93)	69 (5.43)	102 (8.07)	167 (13.23)	
Model 1	1.42 (1.29–1.58)	1.84 (1.54–2.19)	Reference	1.72 (1.11–2.68)	2.48 (1.50–4.12)	4.28 (2.45–7.50)	< 0.001
Model 2	1.37 (1.23–1.53)	1.72 (1.43–2.08)	Reference	1.64 (1.04–2.59)	2.24 (1.33–3.78)	3.89 (2.17–6.96)	< 0.001
aPP, mmHg			PP < 57	57 ≤ PP < 66	66 ≤ PP < 77	PP ≥ 77	
CKD incidence, *n* (*n* per 1000 person years)	376 (7.38)		55 (4.29)	76 (5.96)	112 (8.83)	133 (10.50)	
Model 1	1.02 (1.01–1.03)	1.37 (1.22–1.53)	Reference	1.36 (0.96–1.94)	1.99 (1.41–2.81)	2.71 (1.88–3.91)	< 0.001
Model 2	1.03 (1.02–1.04)	1.51 (1.32–1.72)	Reference	1.65 (1.14–2.38)	2.59 (1.78–3.78)	3.82 (2.52–5.79)	< 0.001

*aPWV* aortic pulse wave velocity, *ecfPWV* estimated carotid-femoral pulse wave velocity, *aPP* aortic pulse pressure

Model 1 is adjusted for age, sex, ethnicity, body mass index, vitamin D treatment group, aortic mean arterial pressure and eGFR measured by creatinine

Model 2 is adjusted for the variables in Model 1 plus lifestyle factors (tobacco smoking, alcohol drinking, vigorous physical activity), medications (antihypertensives, diabetes, lipid-lowering), diabetes/prediabetes, history of cardiovascular disease, total cholesterol, high-density lipoprotein cholesterol, New Zealand Index of Deprivation 2013, heart rate

^a^Data for model 1 and model 2 are hazard ratios (95% confidence intervals) for measures of arterial stiffness as a continuous variable

^b^Data are hazard ratios (95% confidence intervals) for measures of arterial stiffness as a continuous variable and are standardised (per 1 SD)

^c^Participant numbers for model 2 total 4745 due to missing data

In model 1, each SD increase in aortic pulse wave velocity was associated with a 69% greater risk of incident CKD (sHR, 1.69, 95% CI 1.45–1.97), estimated carotid-femoral pulse wave velocity was associated with an 84% greater risk of incident CKD (sHR 1.84, 95% CI 1.54–2.19) and aortic pulse pressure with a 37% greater risk of incident CKD (sHR 1.37, 95% CI 1.22–1.53) after adjusting for age, sex, ethnicity, BMI, vitamin D treatment group, aortic mean arterial pressure and eGFR. The sHRs remained significant in model 2, which also adjusted for lifestyle factors, medications, diabetes/prediabetes, history of CVD, NZDep2013, heart rate, total cholesterol and HDL-C.

**Fig. 1:**
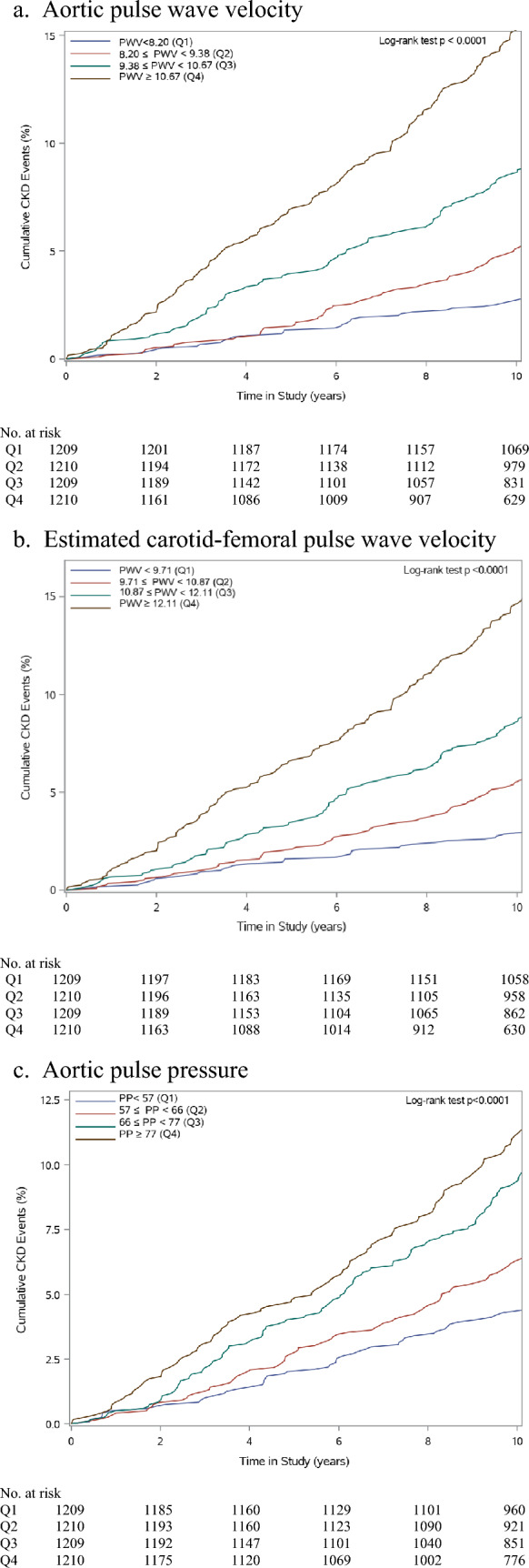
Cumulative incidence of chronic kidney disease (CKD) by arterial stiffness quartiles (1–4); *PWV* pulse wave velocity, *PP* pulse pressure

Figure 1 shows that the cumulative incidence of CKD increased with each increasing arterial stiffness quartile. In model 1, the risk of incident CKD was, compared to the first quartile, higher in the fourth quartile of aortic pulse wave velocity (HR 4.72, 95% CI 2.69–8.27), estimated carotid-femoral pulse wave velocity (HR 4.28, 95% CI 2.45–7.50) and aortic pulse pressure (HR 2.71, 95% CI 1.88–3.91). These associations remained significant when we adjusted for the additional covariates in model 2. There was a significant positive trend for the quartiles of all measures of arterial stiffness for both model 1 and model 2.

Applying aortic pulse wave velocity in model 1, aortic mean arterial pressure (HR 0.98, 95% CI 0.97–0.99), sex (HR for men 2.74, 95% CI 2.17–3.46), ethnicity [European/other, reference; Māori (HR 2.45, 95% CI 1.63–3.68); Pacific (HR 2.67, 95% CI 1.91–3.75); South Asian (HR 3.76, 95% CI 2.43–5.83)], BMI (HR 1.09, 95% CI 1.07–1.11) and eGFR (HR 0.92, 95% CI 0.91–0.93) were all associated with an increased risk of incident CKD (Supplementary Information, Table S2). These associations remained significant when aortic pulse wave velocity was substituted for estimated carotid-femoral pulse wave velocity or aortic pulse pressure. After adjusting for the additional covariates in model 2, all associations, for all models, apart from South Asian for aortic pulse wave velocity and aortic pulse pressure, remained significant. In addition, in model 2, tobacco smoking, vigorous physical activity, NZDep2013, CVD, diabetes and medications (antihypertensives, diabetic) were also associated with incident CKD.

### Sensitivity analysis

We undertook sensitivity analysis where participants with a baseline eGFR < 60 ml/min per 1.73 m^2^ (*n* = 759) were excluded from the analysis (Supplementary Information, Table S3). For both models, when arterial stiffness was measured by aortic pulse wave velocity and estimated carotid-femoral pulse wave velocity, the risk of incident CKD was slightly higher than for when people with a baseline eGFR < 60 ml/min per 1.73 m^2^ were also included (for model 1: sHR 1.74, 95% CI 1.40–2.17; sHR 1.89, 95% CI 1.47–2.44, respectively) and was substantively the same for aortic pulse pressure. There was also a significant positive trend for the quartiles of all arterial stiffness measures for both models.

## Discussion

In a large population-based study, with a follow-up of 10.5 years, higher arterial stiffness, as measured by aortic pulse wave velocity, estimated carotid-femoral pulse wave velocity and aortic pulse pressure, was associated with an increased risk of developing CKD. Further, our results showed that as the quartile of each arterial stiffness measure increased so did the risk of incident CKD.

To our knowledge, this is the first large population study that has investigated the association of arterial stiffness with incident CKD using oscillometrically-measured arterial stiffness. Our study findings are consistent with other large-scale population studies that have found positive associations between arterial stiffness and incident CKD. Those studies which employed carotid-femoral pulse wave velocity include the *Framingham Heart Study* [8] (sHR 1.17, 95% CI 1.01–1.35) and the *Health ABC Study* [9] (incident rate ratio per doubling of carotid-femoral pulse wave velocity 1.42, 95% CI 1.12–1.81). Positive associations were also found where arterial stiffness was measured utilising brachial-ankle pulse wave velocity [10] (odds ratio per m/s higher pulse wave velocity 1.36, 95% CI 1.09–1.70) and heart-ankle pulse wave velocity [11] (sHR 1.12, 95% CI 1.05–1.18).

In contrast, no relationship was found between carotid-femoral pulse wave velocity and incident CKD in the *Rotterdam Study* [13] (relative risk per SD increase in pulse wave velocity 1.05, 95% CI 0.99–1.31) or in the *AGE-RS Study* [12] (odds ratio per m/s higher pulse wave velocity 1.16, 95% CI 0.84–1.61). However, the *Rotterdam Study* [13] may have been subject to selection bias having pulse wave velocity data for only 2665 out of 3666 participants, while the *AGE-RS Study* [12] suffered from attrition bias after losing 310 out of 940 participants to follow-up. These studies, aside from the *Health ABC Study* [9], had mainly ethnically homogeneous populations and were unable to report results by ethnicity.

Māori and Pacific in this study had a higher risk of incident CKD than their European/other counterparts. In addition to having the highest prevalences of socio-economic deprivation in New Zealand, these people also have the highest prevalences of obesity, and hence diabetes and CVD [27]. This in turn is a large driver of the high CKD prevalence in this population. Māori and Pacific, however, still had higher risk of CKD in our analysis when we controlled for NZDep2013, BMI, physical activity, diabetes and CVD risk factors. Although diabetic nephropathy is the major cause of CKD in Māori and Pacific people, there is increasing evidence that there may be a familial or ethnic predisposition to CKD that is not due to diabetes or hypertension [28, 29].

The kidney is a high blood flow organ with low resistance vascular beds. Increased arterial stiffness exposes the smaller arteries of the kidney to increased pulsatile stress, possibly resulting in damage to endothelial and vascular smooth muscle cells [5]. Capillaries of the glomerulus, which are situated between afferent and efferent arterioles, may be particularly susceptible to damage, as the efferent arterioles have greater resistance than the afferent arterioles which leads to high pulsatile pressure in the glomerulus [5].

Our study has certain strengths. First, we had a long follow-up period. Second, as our study also included Māori, Pacific peoples and South Asian ethnicities, our results were generalizable to those populations and not just Europeans. Third, as outcomes were ascertained from hospital discharge registers, there was no loss to follow-up of participants.

This study has some limitations. First, as we did not clinically assess CKD, we may not have excluded all people with CKD from our initial study cohort. We did, however, control for eGFR. Further, in population studies it is widely accepted that CKD can be established with an eGFR < 60 ml/min per 1.73 m^2^ [30]. When we removed people at baseline who fit this definition of CKD, our conclusions remained unaltered. Second, as we had to rely on hospitalisations to identify CKD, not all people who developed CKD would have been identified. Third, as the study is observational, we cannot be completely sure of causality.

To combat the global prevalence of CKD, and as people with this condition are generally asymptomatic, new approaches to the identification and prevention of CKD are needed. We found that arterial stiffness, as measured by aortic pulse wave velocity, estimated carotid-femoral pulse wave velocity and aortic pulse pressure, was positively associated with future CKD development. Arterial stiffness, which can be easily and accurately measured, could be used as a tool in clinical practice (as part of routine BP measurement and diabetes monitoring) to help identify people at risk of CKD.

## Supplementary Information

Below is the link to the electronic supplementary material.

Supplementary file1 (PDF 152 KB)

## Data Availability

Participants gave consent for data to be available only to the study research team
