# Supplementary material for: Arterial stiffness and incident chronic kidney disease: a large population-based cohort study
Source: J Nephrol. 2024 May 29;37(5):1241–50. doi: 10.1007/s40620-024-01968-x (PMC11405492; doi:10.1007/s40620-024-01968-x)
Supplement: Supplementary file 1 — Supplementary file1 (PDF 152 KB) [file 40620_2024_1968_MOESM1_ESM.pdf]

Supplementary Information for

Journal of Nephrology

Arterial stiffness and incident chronic kidney disease: a large population-based cohort study

Angela L Beros, John D Sluyter, Alun D Hughes, Bernhard Hametner, Siegfried Wassertheurer, Robert KR Scragg

Corresponding author:

Prof Robert Scragg
School of Population Health, University of Auckland, Auckland, New Zealand
Email: r.scragg@auckland.ac.nz

Table of Contents

Table S1 Ministry of Health outcomes by clinical coding system codes (ICD-10 Codes: sixth, eight and eleventh editions) 2
Table S2. Incidence and hazard ratios of chronic kidney disease in relation to the total sample for all measures of arterial stiffness[a] 3
Table S3. Incidence and hazard ratios of chronic kidney disease (CKD) in relation to the total sample and quartiles of baseline arterial stiffness measures, excluding participants with baseline eGFR < 60 ml/min per $1.73m^2$.......... 4

Table S1. Ministry of Health outcomes by clinical coding system codes (ICD-10 Codes: sixth, eight and eleventh editions)

Chronic kidney disease outcomes	ICD-10 Code[a,b]
Chronic kidney disease	N18 (more specifically: N18.1 to N18.5, N18.9)
Diabetic kidney disease	E09.2 to E11.2 and E13.2 (more specifically: E09.21, E09.29, E10.21, E10.22, E10.29, E11.21, E11.22, E11.29, E13.21, E13.22, E13.29)
Other kidney disease	N19, I12.0, I12.9, I13.0, I13.1, I13.2, R77.0, R80
Diabetes outcomes	
Impaired glucose regulation (sixth edition), Intermediate hyperglycaemia (eight and eleventh Edition)	E09 (more specifically: E09.2 to E09.9)
Type 1 diabetes mellitus	E10 (more specifically: E10.0 to E10.9)
Type 2 diabetes mellitus	E11 (more specifically: E11.0 to E11.9)
Other specified diabetes mellitus	E13 (more specifically: E13.0 to E13.9)

a The Ministry of Health version of the ICD codes utilized only the sixth, eight and eleventh editions of these codes for our study period

b The following subdivisions are used with E09, E10, E11 .0, with coma; .1, with ketoacidosis; .2, with renal complications; .3 with ophthalmic complications; .4, with neurological complications; .5, with peripheral circulatory complications; .6 with other specified complications; .7, with multiple complications; .8, with unspecified complications; .9 without complications

Table S2. Incidence and hazard ratios of chronic kidney disease in relation to the total sample for all measures of arterial stiffness[a]

	aPWV		ecfPWV		aPP	
	Model 1	Model 2	Model 1	Model 2	Model 1	Model 2
Number, n[b]	4838	4707	4838	4707	4838	4707
aPWV, m/s	1.35 (1.24 to 1.48)	1.31 (1.19 to 1.44)	-	-	-	-
ecfPWV, ms	-	-	1.42 (1.29 to 1.58)	1.37 (1.23 to 1.53)	-	-
aPP mmHg	-	-	-	-	1.02 (1.01 to 1.03)	1.02 (1.02 to 1.04)
Mean arterial pressure, aortic, mmHg	0.98 (0.97 to 0.99)	0.99 (0.98 to 1.00)	0.97 (0.96 to 0.98)	0.98 (0.97 to 0.99)	0.98 (0.97 to 0.99)	0.98 (0.97 to 0.99)
Age, ≥ 65 y	Reference	Reference	Reference	Reference	Reference	Reference
Age, < 65 y	1.02 (0.72 to 1.45)	1.14 (0.79 to 1.63)	1.10 (0.77 to 1.58)	1.21 (0.84 to 1.75)	0.61 (0.46 to 0.81)	0.76 (0.57 to 1.02)
Women	Reference	Reference	Reference	Reference	Reference	Reference
Men	2.74 (2.17 to 3.46)	2.75 (2.11 to 3.59)	2.70 (2.13 to 3.41)	2.70 (2.07 to 3.52)	3.27 (2.59 to 4.13)	3.30 (2.52 to 4.31)
Ethnicity						
European/other	Reference	Reference	Reference	Reference	Reference	Reference
Māori	2.45 (1.63 to 3.68)	1.83 (1.17 to 2.86)	2.42 (1.61 to 3.63)	1.84 (1.17 to 2.87)	2.28 (1.51 to 3.42)	1.74 (1.11 to 2.72)
Pacific	2.67 (1.91 to 3.75)	2.00 (1.33 to 3.00)	2.63 (1.88 to 3.68)	1.94 (1.29 to 2.91)	2.15 (1.54 to 3.01)	1.69 (1.13 to 2.52)
South Asian	3.76 (2.43 to 5.83)	1.58 (0.98 to 2.56)	3.99 (2.57 to 6.21)	1.65 (1.02 to 2.67)	3.28 (2.12 to 5.09)	1.33 (0.82 to 2.14)
Body mass index, kg/m^2	1.09 (1.07 to 1.11)	1.04 (1.02 to 1.07)	1.09 (1.07 to 1.11)	1.04 (1.02 to 1.07)	1.09 (1.07 to 1.11)	1.04 (1.01 to 1.06)
eGFR, ml/min/1.73m^2	0.92 (0.91 to 0.93)	0.93 (0.92 to 0.93)	0.92 (0.91 to 0.93)	0.93 (0.92 to 0.93)	0.92 (0.91 to 0.92)	0.92 (0.91 to 0.93)
Vitamin D supplementation, no	Reference	Reference	Reference	Reference	Reference	Reference
Vitamin D supplementation, yes	0.89 (0.73 to 1.09)	0.84 (0.68 to 1.04)	0.89 (0.72 to 1.09)	0.82 (0.67 to 1.02)	0.91 (0.74 to 1.12)	0.86 (0.69 to 1.06)
Tobacco smoker, past or never	-	Reference	-	Reference	-	Reference
Tobacco smoker, current	-	1.76 (1.12 to 2.77)	-	1.74 (1.11 to 2.74)	-	1.64 (1.04 to 2.57)
Alcohol drinker, past or never	-	Reference	-	Reference	-	Reference
Alcohol drinker, current	-	1.11 (0.82 to 1.50)	-	1.08 (0.80 to 1.47)	-	1.14 (0.84 to 1.55)
Vigorous physical activity, ≤ 2hrs per week	-	Reference	-	Reference	-	Reference
Vigorous physical activity, > 2hrs per week	-	0.75 (0.59 to 0.94)	-	0.77 (0.61 to 0.97)	-	0.70 (0.56 to 0.88)
NZDep2013, levels 1-10	-	1.05 (1.01 to 1.09)	-	1.05 (1.01 to 1.09)	-	1.04 (1.00 to 1.09)
Cardiovascular disease, no	-	Reference	-	Reference	-	Reference
Cardiovascular disease, yes	-	1.56 (1.25 to 1.96)	-	1.51 (1.20 to 1.89)	-	1.58 (1.27 to 1.98)
Diabetes/prediabetes, no	-	Reference	-	Reference	-	Reference
Diabetes/prediabetes, yes	-	2.90 (1.88 to 4.45)	-	2.91 (1.89 to 4.47)	-	2.89 (1.88 to 4.45)
Medications						
Antihypertensives, no	-	Reference	-	Reference	-	Reference
Antihypertensives, yes	-	1.58 (1.24 to 2.02)	-	1.61 (1.26 to 2.06)	-	1.57 (1.23 to 2.01)
Diabetic, no	-	Reference	-	Reference	-	Reference
Diabetic, yes	-	1.74 (1.13 to 2.70)	-	1.79 (1.15 to 2.77)	-	1.67 (1.08 to 2.58)
Lipid lowering, no	-	Reference	-	Reference	-	Reference
Lipid lowering, yes	-	0.96 (0.76 to 1.21)	-	0.98 (0.78 to 1.24)	-	0.90 (0.71 to 1.14)
Total cholesterol, mmol/L	-	0.87 (0.76 to 1.00)	-	0.87 (0.76 to 1.00)	-	0.84 (0.73 to 0.95)
HDL cholesterol, mmol/L	-	1.21 (0.86 to 1.71)	-	1.22 (0.87 to 1.71)	-	1.27 (0.91 to 1.78)
Heart rate, bpm	-	1.01 (1.00 to 1.02)	-	1.01 (1.00 to 1.02)	-	1.02 (1.01 to 1.03)

aPWV indicates aortic pulse wave velocity; ecfPWV indicates estimated carotid-femoral pulse wave velocity; aPP indicates aortic pulse pressure; NZDep 2013, New Zealand Index of Deprivation 2013

[a] Data for Model 1 and Model 2 are hazard ratios (95% confidence intervals) for measures of arterial stiffness as a continuous variable

[b] Participant numbers for Model 2 total 4707 due to missing data

Table S3. Incidence and hazard ratios of chronic kidney disease (CKD) in relation to the total sample and quartiles of baseline arterial stiffness measures, excluding participants with baseline eGFR < 60 ml/min per 1.73m^2

	Total sample[a]	Total sample[b]	Quartile of arterial stiffness – Hazard ratios (95%CI)				P for trend
Participants, n[c]	4079		1019	1021	1020	1019	
aPWV			PWV < 8.04	8.04 ≤ PWV < 9.19	9.19 ≤ PWV < 10.38	PWV ≥ 10.38	
CKD incidence, n (n per 1,000 person years)	189 (4.60)		18 (1.64)	40 (3.72)	52 (4.89)	79 (7.43)	
Model 1	1.39 (1.22 to 1.59)	1.74 (1.40 to 2.17)	Reference	2.66 (1.48 to 4.76)	4.15 (2.11 to 8.16)	7.34 (3.49 to 15.45)	<0.001
Model 2	1.39 (1.20 to 1.60)	1.73 (1.36 to 2.20)	Reference	3.01 (1.62 to 5.59)	4.07 (1.98 to 8.35)	7.19 (3.23 to 16.02)	<0.001
ecfPWV			PWV < 9.56	9.56 ≤ PWV < 10.68	10.68 ≤ PWV < 11.86	PWV ≥ 11.86	
CKD incidence, n (n per 1,000 person years)	189 (4.60)		23 (2.10)	36 (3.35)	53 (4.97)	77 (7.25)	
Model 1	1.47 (1.26 to 1.71)	1.89 (1.47 to 2.44)	Reference	1.92 (1.09 to 3.40)	3.22 (1.67 to 6.20)	5.65 (2.65 to 12.06)	<0.001
Model 2	1.46 (1.24 to 1.73)	1.88 (1.43 to 2.48)	Reference	1.69 (0.94 to 3.04)	2.53 (1.27 to 5.06)	4.62 (2.08 to 10.28)	<0.001
aPP			PP < 57	57 ≤ PP < 66	66 ≤ PP < 75	PP ≥ 75	
CKD incidence, n (n per 1,000 person years)	189 (4.60)		24 (2.22)	41 (3.81)	55 (5.13)	69 (6.47)	
Model 1	1.02 (1.01 to 1.03)	1.35 (1.15 to 1.59)	Reference	1.67 (1.00 to 2.79)	2.40 (1.43 to 4.02)	3.41 (1.98 to 5.88)	<0.001
Model 2	1.03 (1.01 to 1.04)	1.45 (1.20 to 1.75)	Reference	2.19 (1.25 to 3.81)	3.38 (1.88 to 6.07)	5.45 (2.85 to 10.43)	<0.001

aPWV indicates aortic pulse wave velocity; ecfPWV, estimated carotid-femoral pulse wave velocity; aPP, aortic pulse pressure

Model 1 is adjusted for age, sex, ethnicity, body mass index, vitamin D treatment group, aortic mean arterial pressure and eGFR measured by creatinine

Model 2 is adjusted for the variables in Model 1 plus lifestyle factors (tobacco smoking, alcohol drinking, vigorous physical activity), medications (antihypertensives, diabetes, lipid-lowering), diabetes/prediabetes, history of cardiovascular disease, total cholesterol, high-density lipoprotein cholesterol, New Zealand Index of Deprivation 2013, heart rate

[a] Data for Model 1 and Model 2 are hazard ratios (95% confidence intervals) for measures of arterial stiffness as a continuous variable

[b] Data are hazard ratios (95% confidence intervals) for measures of arterial stiffness as a continuous variable and are standardised (per 1 SD)

[c] Participant numbers for Model 2 total 4009 due to missing data
